# Experience from a pilot point-of-care CD4 enumeration programme in Kenya

**DOI:** 10.4102/ajlm.v5i2.439

**Published:** 2016-10-17

**Authors:** Matilu Mwau, Mamo Umuro, Collins O. Odhiambo

**Affiliations:** 1 Kenya Medical Research Institute, Nairobi, Kenya; 2 National Public Health Laboratory Services, Nairobi, Kenya

## HIV situation in Kenya

Kenya is a HIV ‘high burden’ county in Africa with both a generalised and a concentrated epidemic within its population of 47 million.^1^ HIV prevalence reached a peak of 10.5% in 1995–1996, but declined to approximately 6.0% in 2013.^2^ The number of people living with HIV is estimated to have increased from about 1.2 million in 2009 to 1.4 million in 2013.^3^ This number is projected to continue increasing due to improved survival attributable to antiretroviral therapy programmes. The total number of new HIV infections is estimated to have declined by about 15.0% over the last five years from about 116 000 in 2009 to around 100 000 in 2013.^3^ Over the last five years, the number of annual AIDS-related deaths has declined, from about 85 000 in 2009 to 58 000 in 2013. The prevalence of HIV in infants has also been declining, with numbers dropping from around 14.4% in 2007 to 8.8% in 2015. This decline is partly due to a robust prevention of mother-to-child transmission programme. It is estimated that 100 000 infants are born to HIV-positive mothers every year. Approximately 60 000 children are on treatment for HIV. However, only 50.0% of infants receive a timely virologic test and antiretroviral therapy coverage is suboptimal, which can be attributed to limited access to diagnosis.^4^

## Laboratory infrastructure in support of HIV care and treatment in Kenya

For adults, counselling services and HIV testing using rapid test strips are widely available.^3^ Up to 83% of women and 71% of men report having tested at least once in their lifetime. Access to testing is not uniform across the country, with the northern parts (mainly arid, underpopulated and low HIV-prevalence areas) reporting less than 10% testing.^3^

Following the ‘Test and Treat’ guidelines released by the World Health Organization in 2014,^5^ CD4 testing is on the decline. There are approximately 170 BD FACSCount™ machines in Kenya, and fewer than 30 BD FACSCalibur™ machines (personal communication, NPHLS). These are concentrated mainly in highly-populated parts of the country. The total number of tests for CD4 done in 2015 was only 439 443, down from more than 800 000 a year earlier. It is likely that some CD4 testing demand will persist even after ‘test and treat’ is adopted, mainly driven by demand from clinicians and for management of cryptococcal meningitis and other opportunistic infections.

Early infant diagnosis (EID) testing began in 2006 and is offered at seven national laboratories.^4^ The laboratories conduct 95% of all the testing on a referral basis. The monthly workload is 5000 tests, and has been steady. This volume represents only 50% of the infants who should be tested, due to suboptimal access, partly as a result of the centralised laboratory referral system. The Abbot *m*2000sp™ and Roche COBAS^®^ AmpliPrep/COBAS^®^ TaqMan^®^ systems are in widespread use, running on medium throughput. Viral load testing was available as a private service in Kenya until October 2014, when the national programme decided to roll out the service free of charge in the public sector. There are seven laboratories that form part of the viral load testing network, with a combined total of 17 Roche and 16 Abbott instruments. Between them, up to 900 000 viral load tests will be done in 2016. The demand, however, is estimated to be approximately 3.4 million viral load tests over the next three years.

## Quality assurance framework and policy for HIV lab and point-of-care testing

To help improve access to viral load and EID testing, the Ministry of Health encouraged evaluations of new technologies as they become available. It has also developed a point-of-care (POC) testing policy to guide the implementation of POC testing to support laboratory practice and bring services closer to patients. A POC technical working group comprising several stakeholders, including the Elizabeth Glaser Pediatric AIDS Foundation, the Clinton Health Access Initiative and the United Nations Children’s Fund, is overseeing the implementation.

Currently, the National Public Health Laboratories Services, in collaboration with key stakeholders, is putting together a national POC roadmap that addresses the introduction and scale-up of POC testing in the existing landscape. The Kenya Medical Research Institute has been evaluating technologies as they become available in order to determine their suitability for use in Kenya.^6,7,8^ Currently, EID and viral load POC testing devices are under evaluation, with results expected in late 2016.

**FIGURE 1:**
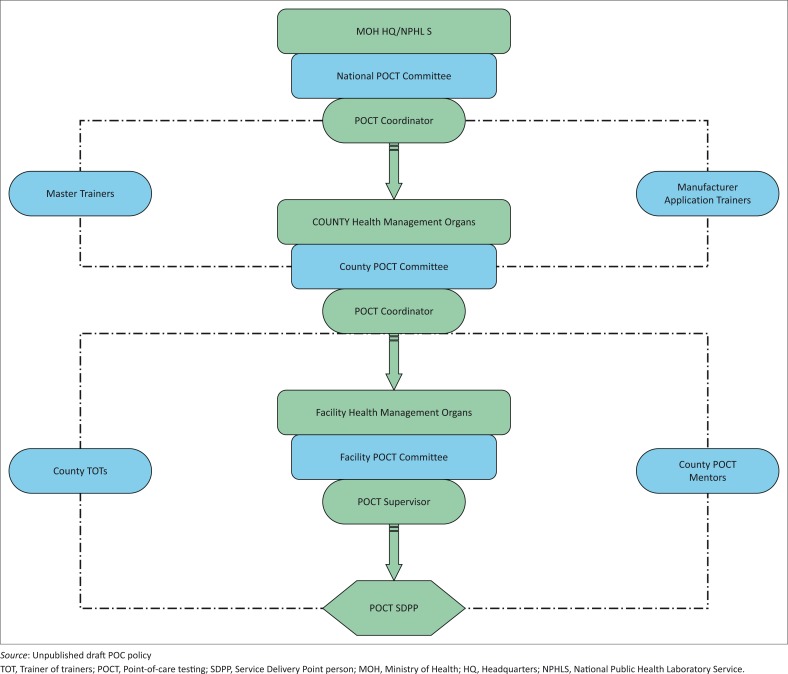
Kenya point-of-care quality assurance programme management structure.

Guidelines for POC technology evaluation and adoption were developed and launched in 2014. The national coordinator for POC evaluations is the National Public Health Laboratories Services, but there are sub-coordinators in all 47 counties of Kenya (Figure 1). The coordinators are mainly involved in supportive supervision. A national laboratory technical advisory committee develops policies and guidelines for POC testing. Other components of the guideline address training, competency assessment and certification of end-users. The guideline also addresses the selection and assessment criteria for POC sites. The Ministry of Health and implementing partners have started discussions on mapping of viral load and EID to identify gaps that POC devices could help fill.

## Existing quality assurance programmes

Kenya has a national quality assurance programme that has been pursuing laboratory accreditation through the Strengthening Laboratory Management Towards Accreditation approach.^6^ The programme also supports External Quality Assessment (EQA), training and registration of users, and harmonisation of protocols and standards.

EQA occurs three times a year in Kenya through an intermediate model involving both local and international EQA providers. International panels are sourced from Human Quality Assessment Services, Public Health Agency of Canada (QASI), the US Centers for Disease Control and Prevention, and the World Health Organization, depending on the test. The EQA panels are distributed to county hubs for redistribution to the testing sites. National panels, including dried tube specimens, split samples, rechecking and internal controls, are procured centrally. The national programme also supports validation of new tests/equipment, certification of personnel and routine maintenance of equipment. Routine supervision is decentralised.

All seven central molecular testing laboratories participate in the US Centers for Disease Control and Prevention, Atlanta, proficiency testing for viral load, EID and HIV drug resistance testing. Additionally, a subset (4/7) of the central testing labs participate in a quarterly inter-laboratory EQA programme, with all expected to participate during 2016. Samples are processed between the participating laboratories and results are discussed every quarter to enhance quality. It is conceivable that there will be three cycles of EQA annually for EID and viral load when the appropriate POC testing devices become available.

## Outcomes

**FIGURE 2:**
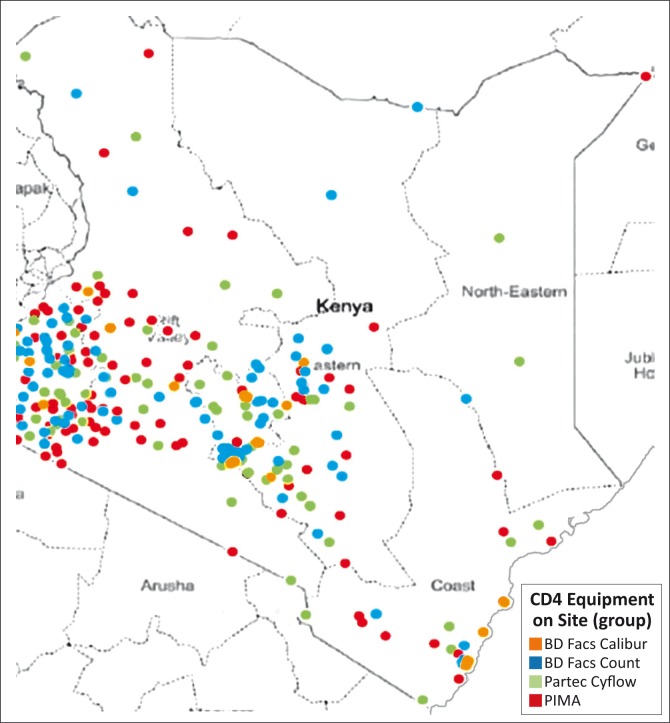
CD4 device mapping in Kenya. A total of 298 functioning CD4 instruments were clustered across 47 counties.

The POC testing pipeline has only had a few technologies that have reached clinical evaluation, and of those, the Alere Pima™ and BD FACSPresto™ have shown the biggest potential for use. Following satisfactory evaluation findings,^8^ Kenya deployed 48 Alere Pima devices across 34 of its 47 counties in 2014, including four devices at sites supported by Médecins Sans Frontières (Figure 2). Training for an additional 62 sites was completed in March 2015, which increased the coverage of POC CD4 devices in the country to 44 out of 47 counties. Overall, 96 sites have a total of 119 Pima devices. At least three personnel from each testing site were trained on the use of the POC testing device.

Site selection was based on a national map of existing CD4 sites and extensive discussion between regional implementing partners and individual County Health Management teams who oversee all health-related issues in the county. This process led to the generation of a list of 150 facilities that fit the site selection criteria agreed upon by the POC Technical Working Group, such as total patient volumes per health facility, distance travelled to central testing hubs, and the total throughput of the Pima device. These criteria were used to generate a score for each site that was eligible for Pima placement. In addition, qualitative criteria, such as security and availability of personnel, were considered when selecting placements for the initial 44 devices from the 150 sites generated. The same site selection methodology was applied for the 62 additional Pima devices that were deployed in early 2015.

The Ministry of Health has enacted a law passed by parliament that recommends device leasing (as opposed to purchase) for all medical diagnostic services in the country. In this model, the device belongs to the manufacturer during its usage period. The manufacturer is responsible for the maintenance of the device. The cost of the cartridge, service and maintenance is bundled and packaged together with the cost per test.

In 2014, trainings were conducted using the super-user training model and included the training of County Laboratory Coordinators, implementing partners, and a team from the National HIV Reference Laboratory. However, the quality of training that cascaded down through training-of-trainers was poor and therefore required follow-up for on-site re-training by the Ministry of Health, supported by implementation partners. In 2015, trainings focused on an end-user training model.

While most facilities were able to begin testing within one to two weeks after the super-user training, there was delayed end-user/facility-based training in some facilities due to conflicting commitments by either the County Laboratory Coordinator or the implementing partner. The lessons learned from 2014 have been applied to the 2015 training curriculum, such as the switch from super-user training to end-user training.

Follow-up visits after the initial training and implementation of the 44 Pima devices showed that most POC testing sites adapted their clinical workflow around the availability of onsite CD4 testing. Nearby satellite sites are now redirecting their samples to Pima POC testing sites. Using this approach, the 119 Pima devices performed an average of 2432 tests per month, or 6.5% of all CD4 tests done in the country. The BD FACSPresto was rolled out at 13 sites, for an average of 818 tests a month.

At POC testing sites, 71.5% of patients received same day results, while post-implementation assessments have shown a reduction in time to initiation on antiretroviral therapy from two months^8^ to 27.5 days at POC testing sites.^6^ Additionally, 11.7% of all eligible patients were initiated on antiretroviral therapy on the same day they were tested.

## Challenges and lessons learnt

The first phase of implementation of POC CD4 testing devices in Kenya revealed a number of challenges:

1. Testing errors resulted in wastage of a significant number of test cartridges for the Pima device. In 2014, the error rate was 7.6% of all tests done, while in 2015, this rate increased to 13.6% in the first two quarters of the year. The maximum pass rate for the EQA system over 40 rounds was only 82.0%.
2. Lack of reporting on commodity consumption resulted in stock-outs at some sites and the delay in issuing new control beads for the Pima machines. Machine downtimes reduced overall access to the service.
3. Movement of staff within counties while organisational structures were finalised meant that some of the staff trained as super-users were transferred to new regions and could no longer provide support to the Pima POC testing sites in the region in which they were trained.
4. Other competing engagements of some county super-users meant that they were unable to provide consistent mentorship or support to the users they trained.
5. Lack of internet in remote regions meant data transmission to the national database was erratic.

### Lessons learnt

POC technologies for HIV have the potential to improve access to diagnosis and to reduce time to result or to initiation of treatment. Kenya, being a high HIV burden country, is keen to adopt technologies that offer the most promise and is therefore constantly evaluating them.

During the rollout of the Alere Pima and BD FACSPresto systems, some lessons have been learnt that can guide future adoption of POC testing for viral load and EID. The rate of testing errors, and the causes, does not differ markedly from what others have reported.^10^ Errors attributed to the user can be mitigated by supportive supervision and trainings; device errors can be reduced through preventative maintenance, while errors attributed to the sample can be managed through training of end-users.

The POC CD4 testing programme was hampered by poor reporting on commodity consumption. The net effect was a lack of supplies, leading to avoidable machine downtime. In our experience, supportive supervision improves reporting. An even more effective solution is automating the reporting by means of digital transmission systems.^4,10,11^ This requires that POC devices come with preinstalled Global System for Mobile Communications or general Packet Radio Service capability.

**FIGURE 3:**
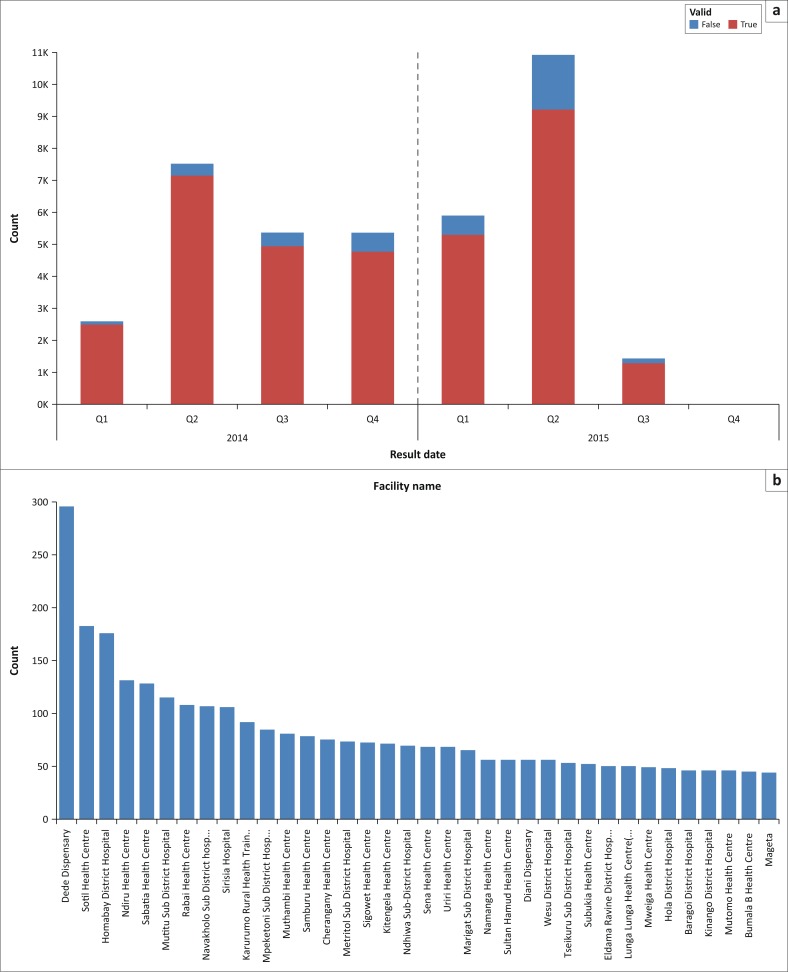
(a) CD4 Pima™ POC testing trends 2014–2015 error rates; (b) Drill down to sites with high error rates: top 36 sites in Kenya contributing to 75% of the total error rates.

After each EQA cycle, the National HIV Reference Laboratory and supporting partners need to conduct intervention meetings with participating sites with unsatisfactory reports to support improvement of performance and enhance quality service delivery (Figure 3). Kenya is undertaking a costing exercise for quality assurance for POC testing, (wherein 3 members of the Kenya Medical Research Institute have been undergoing a series of trainings, facilitated by the London School of Hygiene and Tropical Medicine and the International Diagnostics Centre, to build their capacity to cost quality assurance models), but regardless of the findings, the Ministry of Health will need to identify additional support from implementing partners to establish local production of EQA panels.

### Conclusion

Kenya has been very engaged with the evaluation and implementation of POC testing and pursues the highest quality of care for Kenyans through quality diagnostics. The MOH is continuously expanding quality assurance programmes with an emphasis on the accreditation of laboratories and plans for local production of external quality assurance panels.

Implementing partners can continue to work with the Ministry of Health to mitigate further risk and to address the various challenges raised above in some of the following ways: including different end-users in trainings to address the turnover of staff and support trained super-users; and leveraging resources across relevant partners and organisations to ensure that an internet connection is available at every facility to enable online reporting of monthly consumption.

The lessons learned through the implementation of PIMA are guiding the planning for EID and viral load POC; primarily site selection and continuous quality assurance for monitoring and evaluation. The collaboration between implementing partners and Ministry of Health is an important aspect to planning for sustainable quality POC testing.
